# Step-Down of FSH- Dosage During Ovarian Stimulation – Basic Lessons to Be Learnt From a Randomized Controlled Trial

**DOI:** 10.3389/fendo.2021.661707

**Published:** 2021-04-13

**Authors:** Barbara Lawrenz, Carol Coughlan, Laura Melado, Shieryl Digma, Junard Sibal, Alliza Jean, Human M. Fatemi

**Affiliations:** ^1^ In-Vitro-Fertilisation (IVF) Department, ART Fertility Clinics, Abu Dhabi, United Arab Emirates; ^2^ Obstetrical Department, Women’s University Hospital Tuebingen, Tuebingen, Germany; ^3^ Clinical Laboratory, ART Fertility Clinics, Abu Dhabi, United Arab Emirates

**Keywords:** ovarian stimulation, progesterone, reduction of FSH-dosage, systemic FSH level, progesterone elevation

## Abstract

A rise in serum progesterone in the late follicular phase is a well described adverse effect of ovarian stimulation for IVF/ICSI. Previous data suggest, that enhanced gonadotropin stimulation causes progesterone elevation and the incidence of premature progesterone elevation can be reduced by declining gonadotropin dosages. This randomized controlled trial (RCT) aimed to achieve a significant reduction of the progesterone level on the day of final oocyte maturation by a daily reduction of 12.5 IU rec-FSH from a follicle size of 14 mm in a GnRH-antagonist protocol. A total of 127 patients had been recruited (Control group (CG): 62 patients; Study group (SG): 65 patients). Due to drop out, data from 108 patients (CG: 55 patients; SG: 53 patients) were included into the analysis. Patients’ basic parameters, gonadotropin (Gn)-starting dose, total Gn-stimulation dosage, the number of retrieved and mature oocytes as well as in the hormonal parameters on the day of trigger (DoT) were not statistically significantly different. However, through stepwise Gn-reduction of 12.5 IU/day in the SG, there was a statistically highly significant difference in the Gn-stimulation dosage on the day of trigger (p < 0.0001) and statistically significant associations for the DoT-P4-levels with the DoT-FSH-levels for both groups (CG: p = 0.001; SG: p = 0.0045). The herein described significant associations between DoT-P4-levels and DoT-FSH-levels confirm the theory that enhanced FSH stimulation is the primary source of progesterone elevation on the day of final oocyte maturation in stimulated IVF/ICSI cycles. Given the pathophysiologic mechanism of progesterone elevation during ovarian stimulation, the use of an increased FSH step-down dosage should be studied in future RCTs, despite the fact that a step-down approach of daily 12.5 IU rec-FSH did not achieve a significantly reduced progesterone level on the DoT.

**Clinical Trial Registration:** clinicaltrials.gov, identifier NCT03356964.

## Introduction

Ovarian stimulation with gonadotropins significantly alters the endocrine profile during the follicular and luteal phase, as compared to a natural cycle. A well described adverse effect of ovarian stimulation for IVF/ICSI is a premature rise in serum progesterone identified in the late follicular phase. Progesterone elevation (PE) is a common event with a reported incidence of up to 38% (1, 2), and has been extensively researched over the past two decades. Research has focused not only on the impact of PE on the ART-outcome in fresh and frozen embryo transfer cycles, but also on therapeutic strategies to prevent premature rise (3). Despite extensive research and debate surrounding premature PE during ovarian stimulation the etiology and underlying pathophysiology is not completely understood.

Progesterone rise during the late follicular phase of stimulated ART cycles precedes the administration of triggering for final oocyte maturation and is not associated with a premature LH surge, therefore, it does not reflect a “true” luteinization event (1).

Several studies from in-vivo- and in-vitro-data point to the fact that premature progesterone elevation might be triggered by enhanced FSH stimulation (4–7).

FSH is an absolute requirement for preovulatory follicular development and the number of recruited growing follicles is dependent on the duration and magnitude of FSH stimulation (8). In a natural cycle, high FSH levels during the luteo-follicular transition promote the development of a follicular cohort. FSH levels decrease during the mid/late follicular phase, consequently, the follicle with the highest sensitivity for FSH will gain dominance and become the dominant follicle, despite declining FSH-levels (9). Following the FSH window/threshold concept, multifollicular growth in ovarian stimulation cycles for IVF/ICSI is achieved by administration of exogenous gondadotropins on a daily basis to maintain serum FSH levels above the threshold required for multiple follicular development (9, 10). In contrast to a natural cycle, the follicles are subjected to intense and continual FSH stimulation until final oocyte maturation.

The *in-vitro*-study on human ovarian cortical samples and a non-luteinizing FSH-responsive human mitotic granulosa cell line by Oktem et al. (7) provided the molecular evidence for a direct stimulatory effect of FSH on progesterone production. The authors demonstrated clearly that FSH, in addition to its stimulatory effect on the expression of other enzymes required for estrogen synthesis, stimulates 3β-HSD (3β-hydroxysteroid dehydrogenase) expression and progesterone biosynthesis in human granulosa cells and ovarian tissue samples, thus leading to an increase in the conversion of pregnenolone to progesterone. Moreover, the output of progesterone from the samples, stimulated with FSH, was increased in conjunction with rising estradiol levels in a dose-dependent manner.

The data from Oktem et al. (7) are clinically supported by the findings, that ovarian stimulation with the gonadotropin corifollitropin alpha (CFA), which is characterized by peak concentrations two days after the injection and thereafter declining serum CFA-concentrations, resembling a step-down-protocol, leads to a significantly reduced incidence of premature progesterone elevation on the day of final oocyte maturation (6).

Following on from the notion that reduction of FSH-dosage towards the end of ovarian stimulation will lead to a lower incidence of premature progesterone rise, the primary objective of the current study was to evaluate the impact of a very small daily rec-FSH dosage (12.5 IU) reduction during the late follicular phase on the serum progesterone levels on the day of final oocyte maturation. The rationale behind the 12.5 IU reduction dosage was the achieved reduction in progesterone levels which we had seen in our daily clinical experience to prevent progesterone elevation and to avoid possible follicle atresia due to a lack of gonadotropin stimulus. The secondary objective of this study was to evaluate which factors might have an impact on the progesterone levels during ovarian stimulation for IVF/ICSI, thereby providing a better insight into the underlying pathophysiologic mechanism of premature progesterone elevation.

### Material and Methods

This prospective randomized controlled study was performed in ART Fertility Clinic, Abu Dhabi, UAE, between November 2017 and February 2020. Patients with an indication for IVF/ICSI due to primary/secondary infertility, undergoing ovarian stimulation with Rec-FSH/GnRH-antagonist and who consented to take part in this study, were included. The inclusion criteria were an age between 18 and 40 years, a weight of 60 kg up to and including 90 kg, which results in a BMI of 18–32 kg/m2 and a regular menstrual cycle length of 24–35 days. Exclusion criteria were: the presence or history of an endocrine abnormality, abnormal serum biochemistry or hematology, relevant ovarian-, tubal- or uterine-pathology which may adversely affect ovarian stimulation treatment, a history of ovarian hyper response (more than 30 follicles ≥ 11 mm) or ovarian hyperstimulation syndrome (OHSS), polycystic ovary syndrome (PCOS) (11), a history of poor ovarian response, according to the Bologna-criteria and ovarian reserve parameters’ indicating the risk of poor ovarian response (AFC < 5 and AMH < 0,5ng/ml) (12) as well as according to the POSEIDON criteria (13) and endometriosis stage III/IV.

Patients were randomly allocated in a 1:1 ratio to receive the pre-established treatment following a randomization list, performed by an online randomization program. Online-randomization list was performed by the study nurse and patients were randomized to the control- or study group after agreeing to take part in the study and prior to commencing ovarian stimulation. For each study participant only one stimulation cycle was included. Due to the nature of the study which required dosage adaptation, patients and their treating physician were not blinded.

In keeping with routine clinical practice, patients were monitored during ovarian stimulation for IVF/ICSI treatment with serial transvaginal ultrasound examinations. Transvaginal scans were performed using a Voluson 6 (GE Healthcare, Milwaukee, WI, USA) ultrasound machine, equipped with a 7–10 MHz two-dimensional transvaginal probe. The patients were asked to empty their bladders and were placed in the lithotomy position. On day 2/3, prior to initiation of stimulation a transvaginal ultrasound was performed to determine the AFC. All follicles with a diameter between 2 and 8 mm in each ovary were recorded and the numbers added to determine the total AFC-count. Follicle size and number were determined during the course of stimulation and on the day of final oocyte maturation by transvaginal ultrasound, as previously described. Follicle size was determined by measuring two orthogonal diameters and the mean value was recorded as follicle size.

Blood samples for this study were taken in addition to routine serum samples used for assessment of ovarian response to stimulation in conjunction with serial ultrasound monitoring of follicular growth. Routinely, blood samples were taken on day 2/3 prior to initiation of stimulation and on each subsequent visit to the clinic during the course of stimulation. This practice included blood tests taken on the day of final oocyte maturation and for the study group also at the start of Gn-dose-reduction. The blood was centrifuged for 10 minutes at 4000 rpm (revolution per minute) and the supernatant was retrieved and frozen at – 21°C. For the measurement of E2, progesterone, FSH and LH, the samples were thawed by keeping them for maximum 90 minutes at room temperature (approximately 20°C - 24°C) and analyzed the same day with the same batch of reagents.

The following data per patient were recorded: age, Body Mass Index (BMI), type of infertility (primary/secondary), cycle length, number of previous pregnancies and miscarriages, years of infertility, number of previous stimulations. On the day of final oocyte maturation, the total number of follicles, the number of follicles between 11 and < 17 and ≥ 17 mm and the cycle day were registered. Moreover, for both groups, the number of stimulation days, the total gonadotropin dose and total amount of GnRH-antagonist required, and the number of retrieved and mature oocytes were recorded. For the study group, the following stimulation parameters were additionally noted: Number of stimulation days until start of Gn-reduction, number of stimulation days with reduced Gn-dosage, Gn-dosage until Gn-reduction, Gn-dosage after Gn-reduction until day of final oocyte maturation, total follicle number and number of follicles ≥ 14 mm at the start of Gn-reduction.

### Ovarian Stimulation Protocol

Ovarian stimulation was performed in Gonadotropin-Releasing-Hormone (GnRH)-antagonist-protocol, using rec-FSH (recombinant Follicle-stimulating-hormone, Gonal f^®^, MERCK-SERONO, Darmstadt, Germany). Stimulation medication dosage was individualized according to the patients’ requirement in accordance with the ovarian reserve parameters (14) and according to our previous experience from daily clinical routine in a mainly dominated Arab population, with the gonadotropins being administered in the evening at 8.00 p.m. commencing on day 2 or 3 of the cycle. To inhibit premature LH surge, daily GnRH – antagonist (Cetrotide^®^ 0.25 mg, MERCK-SERONO, Darmstadt, Germany) was administered from the morning (8:00 am) of day 5 of stimulation and then continued on a daily basis including the day of final oocyte maturation.

#### Control Group

Patients randomized to the control group completed their treatment cycle with a constant stimulation dosage for the entire duration of stimulation, until the criteria for final oocyte maturation were met.

#### Study Group

Patients randomized to the study group started to reduce the gonadotropin dosage daily by 12.5 IU rec-FSH as soon as ≥ 3 follicles ≥ 14 mm were present until the criteria for final oocyte maturation were met.

The follicle-size cut-off value of 14 mm was chosen based on previously published data from coasting studies demonstrating continuous follicle growth from threshold size of approximately 15 mm for an interval even without gonadotrophin support (15, 16). For the control group, as well as for the study group, final oocyte maturation was achieved by administration of either 5.000 IU of hCG or, in case of a risk of ovarian hyperstimulation syndrome (OHSS) (17), with 0.3 mg of GnRH agonist (Triptorelin) as soon as ≥ 3 follicles ≥ 17 mm were present. Oocyte retrieval was carried out 36 hours after administration of the trigger.

In the event of unexpected high or low response requiring dosage adaptation outside the remits of the study protocol, affected patients were excluded from the study.

Blood samples for the serum hormonal measurements were taken 14 – 16 hours after the last gonadotropin injection and 2 – 4 hours after the last GnRH-antagonist injection.

### Hormonal Measurements

#### Progesterone Analysis

ELECSYS^®^ progesterone generation III assay is an electrochemiluminescence immunoassay (ECLISA) which uses sheep monoclonal antibodies due to their higher specificity towards progesterone. The measuring range is 0.159 – 191 nmol/L or 0.05-60 ng/ml. For detection of analytical specificity, cross-reactivities towards other hormones were used with a maximum cross-reactivity of 3.93% towards 11-Deoxycorticosterone and the minimum cross-reactivity of 0.001% towards Danazol (18).

#### Estradiol Analysis

The Elecsys Estradiol III assay employs a competitive test principle using two monoclonal antibodies specifically directed against 17β−estradiol. Endogenous estradiol released from the sample by mesterolone competes with the added estradiol derivative labeled with a ruthenium complexa) for the binding sites on the biotinylated antibody. Results are determined *via* a calibration curve which is instrument specifically generated by 2−point calibration and a master curve provided *via* the reagent barcode or e−barcode.

#### LH analysis

The Elecsys LH assay employs two monoclonal antibodies specifically directed against human LH. The two specific antibodies used recognize particular conformations, with the biotinylated antibodies detecting an epitope constructed from both subunits whereas the antibody with the ruthenium complexa) label detects an epitope from the β−subunit. As a result, the Elecsys LH assay shows negligible cross−reactivity with FSH, TSH, hCG, hGH, and hPL. Results are determined *via* a calibration curve which is instrument-specifically generated by 2−point calibration and a master curve provided *via* the reagent barcode or e−barcode.

#### FSH Analysis

The Elecsys FSH assay employs two different monoclonal antibodies specifically directed against human FSH. Cross−reactivity with LH, TSH, hCG, hGH, and hPL is negligible. Results are determined *via* a calibration curve which is instrument-specifically generated by 2−point calibration and a master curve provided *via* the reagent barcode or e−barcode.

#### AMH Analysis

Serum AMH concentrations were measured by Elecsys^®^ AMH automated assay (for Cobas 601 platform, Roche^®^) for all patients. Imprecision expected from the assay was <5%, as described by the manufacturer; intra-assay and inter-assay co-efficient of variation for Elecsys^®^ AMH automated assay has been reported as 0.5 – 1.4% and 0.7 – 1.9%, respectively.

### Sample Size Calculation

The sample size was calculated to show an estimated difference of progesterone levels of approx. 0.3 ng/ml between study and control group on the day of final oocyte maturation. The estimated difference between the groups was based on our daily clinical findings.

The estimated progesterone values for the control- and the study group were defined according to our daily clinic practice with a mean for the CG (exp): 1.3 ng/ml, and for the mean of the SG (obs): 0.98 ng/ml. Standard deviation (SD)1 or Tolerance: 0.6, SD2: 0.6, Allocation Ratio: 1, Power: 80; Alpha: 5; Method: TwoSample. Analysis without continuity correction: z for 1-power=0.84, z for alpha double sided=1.96, z for alpha single sided=1.64

TWO SAMPLE ANALYSIS: RESULTS for double sided: The sample size required for group1=n1 = 54; the sample size required for group2=n1*allocation ratio=54*1 = 54; The total sample size required N=n1+n2 = 54+54 = 108.

### Statistical Analysis

Normal distribution of the data was verified with Kolmogorov-Smirnoff tests. Since the test of normality rejected the null hypothesis of normality, continuous variables are summarized according to median. Mean values are given as informative purpose. Categorical data is presented in frequency tables and histograms.

Wilcoxon rank sum test was applied using the NPAR1WAY procedure to test for location and scale differences in both groups. Proportions in both groups were compared using the chi square test.

A linear regression analysis was conducted in order to understand the predictivity of FSH to P4 and vice versa. No missing data was found on the final database.

Univariate analyses test was performed with Glimmix to find the predictive factors to P4 and FSH levels. The variables whose P-value was less than 0.20 in univariate analysis were entered in the multivariate analysis. Glimmix procedure was chosen because it fits models with observations that do not all have the same distribution. A multivariable model was implemented to quantify associations between P4, and the different variables grouped in three groups: demographics, stimulation and follicle parameters. The same procedure was applied for the FSH multivariate analysis.

A p value ≤ 0.05 was considered statistically significant if not otherwise specified. SAS^®^Studio Edition was used to perform and analyze the data.

The authors, who are identical with the treating physicians, and the statistician were not blinded to the randomization.

### Ethical Approval and Trial Registration Number

This study was approved by the ethics committee of the IVIRMA Abu Dhabi Fertility Clinic, Abu Dhabi, UAE (approval number: REFA012) (now ART Fertility Clinic Abu Dhabi, UAE) and was registered with clinicaltrials.gov. under the number NCT03356964.

## Results

Between November 2017 and February 2020 March 2020, a total of 127 patients had been recruited for this study, 62 patients for the control group (CG) and 65 patients for the study group (SG). All 127 commenced stimulation on day 2 or 3 (day one of the period was considered when full blood flow started before 9.00 a.m.) of their period with rec-FSH (recombinant FSH, Gonal f^®^, MERCK, Darmstadt, Germany) for ovarian stimulation. During the ovarian stimulation, 7 patients from the CG and 12 patients from the SG were excluded. Reasons for exclusion were: Necessity to increase the Gn-dosage due to unexpected low response (5 patients in CG, 7 patients in SG), treatment cancellation due to personal reasons (2 patients in CG and 2 patients in SG) and 3 patients of the SG administered incorrect Gn-dosages during stimulation. For the final analysis, data of a total of 108 patients (55 patients in CG, 53 patients in SG) were included.

There were no statistically significant differences in the patients’ basic parameters (age, AMH, AFC on day 2 or 3 prior to stimulation start, number of years of infertility and number of previous stimulations, BMI, TSH and the hormonal parameters (estradiol (E2), progesterone (P4), FSH and LH) at the start of stimulation on day 2 or 3 between the patients randomized in CG and in SG. Patients’ characteristics are summarized in **Table 1**.

**Table 1:** Patient’s characteristics.

Parameter	CG (median)	IQR	SG (median)	IQR	p-value
Years of infertility (no)	2	2	2	3	0.373
No of previous stimulations	0	1	0	1	0.457
Age (in years)	29	6	32	7	0.08
AMH (in ng/ml)	3.01	1.8	3.16	1.4	0.627
BMI (in kg/m^2^)	24.8	4.1	25.6	4.4	0.179
Day 2 or 3 AFC (no)	16	7	17	5	0.277
Day 2 or 3 LH (IU)	6.83	2.9	6.45	2.8	0.338
Day 2 or 3 E2 (pg/ml)	45.7	15.4	42.7	10.6	0.179
Day 2 or 3 FSH (IU)	6.53	2.3	6.14	2.1	0.338
Day 2 or 3 P4 (ng/ml)	0.86	0.2	0.96	0.2	0.1799
TSH (in mIU/ml)	1.4	1	1.39	0.7	1

CG, Control group; SG, Study group; E2, estradiol; P4, progesterone; AFC, Antral follicle count; AMH, Anti-Muellerian-Hormone; BMI, Body Mass Index; no. number; IQR, Inter Quartile Range.

The mean starting dose was 215.4 in the CG (range 150 – 450 IU) and 218.4 IU in the SG (range 175 – 375 IU) and median starting dose was 187.5 IU in CG and 225 IU, respectively, which was not statistically significant (p = 0.179). Through the stepwise Gn-reduction of 12.5 IU per day in the SG, starting from the day when the ultrasound scan identified ≥ 3 follicles of ≥ 14 mm, there was a statistically highly significant difference in the median Gn-stimulation dosage on the last day of the stimulation (p < 0.0001). The median total Gn-stimulation dosage was statistically not significant different (CG: 2000 IU, SG: 1987.5 IU, p = 0.846) between the two groups. Median stimulation duration was identical in both groups (10 days, p = 0.45) as well as the total number of follicles on the day of trigger (DoT) (18 follicles in each group, p = 0.73). No statistically significant differences were found in the number of retrieved and mature oocytes (CG: 15/12; SG: 16/12, p = 0.62/0.78, respectively). The hormonal parameters on the DoT were not statistically significantly different. **Table 2** summarizes the stimulation parameters.

**Table 2:** Stimulation parameters.

Parameter	CG (median)	IQR	SG (median)	IQR	p-value
Gn-dosage stimulation start, day 2 or 3 (IU)	187.5	37.5	225.0	37.5	0.1799
Gn-dosage stimulation end, DoT (IU)	187.5	37.5	187.5	50	<.0001**
Total number of stimulation days	10	1	10	1	0.455
DoT no of follicles 11 – 16 mm	9	6	10	6	0.529
DoT no of follicles ≥ 17 mm	5	4	6	4	0.494
DoT total no of follicles	18	8	18	8	0.738
Total Gn dosage (in IU)	2000	562.5	1987.5	462.5	0.846
Total dosage of GnRH antagonist (in mg)	1.5	0.25	1.5	0.25	0.347
No of retrieved oocytes	15	10	16	7	0.622
No of mature oocytes	12	8	12	6	0.783
FOI	0.89	0.47	0.895	0.52	0.271
DoT E2 (pg/ml)	2125	1408	2431	1296	0.565
DoT FSH (IU)	13.4	5.0	12.7	4.8	0.565
DoT LH (IU)	1.2	1.3	1.2	1.5	0.848
DoT P4 (ng/ml)	0.86	0.4	0.97	0.52	0.179

CG, Control group; SG, Study group; Gn, Gonadotropin; DoT, Day of Trigger; IU, International Units; no, number; FOI, Follicle-to-oocyte Index (19); E2, estradiol; P4, progesterone; IQR, Inter Quartile Range; **, highly statistically significant.

For both groups, the evolution of the hormones E2, P4, LH and FSH were followed over the course of the stimulation. For the CG, results are available from the start of GnRH antagonist (DGnstart) and day of trigger (DoT) and for the SG additionally on the day, when the reduction of the gonadotropin dosage was initiated. In the SG, the median number of days with a continuous stimulation dosage was 7 days and 3 days with a reduced stimulation dosage. Median Gn-stimulation dosage was 1500 IU rec-FSH until the start of Gn-reduction and 600 IU rec-FSH after reduction. The median serum FSH-levels, on the start of Gn-reduction were almost identical with the FSH-levels on DoT (12.97 IU/12.67 IU, p = 0.56), whereas the levels of E2 and P4 increased during the supra mentioned period. The median levels of the hormones at the time points are given in **Table 3**.

**Table 3:** Evolution of the hormonal parameters of CG and SG for the timepoints: start of GnRH antagonist, start of Gn-reduction and day of trigger.

	Start of GnRH antagonist (median)	Start of Gn-reduction (median)	DoT(median)	p-value
**CONTROL GROUP**				
**E2 (pg/ml)**	610.25	N/A	2125.0	<.0001
**P4 (ng/ml)**	0.30	N/A	0.86	<.0001
**FSH (IU)**	12.5	N/A	13.44	0.56
**LH (IU)**	1.48	N/A	1.20	0.63
**STUDY GROUP**				
**E2 (pg/ml)**	602.75	1458	2431	<.0001
**P4 (ng/ml)**	0.33	0.60	0.96	<.0001
**FSH (IU)**	12.74	12.97	12.67	0.68
**LH (IU)**	2.28	2.19	1.24	0.039

CG, Control group; SG, Study group; E2, estradiol; P4, progesterone; FSH, Follicle Stimulating Hormone; LH, Luteinising Hormone; IU, International Units; Gn, Gonadotropin; GnRH, Gonadotropin Releasing Hormone; DoT, day of trigger.

**Figure 1:**
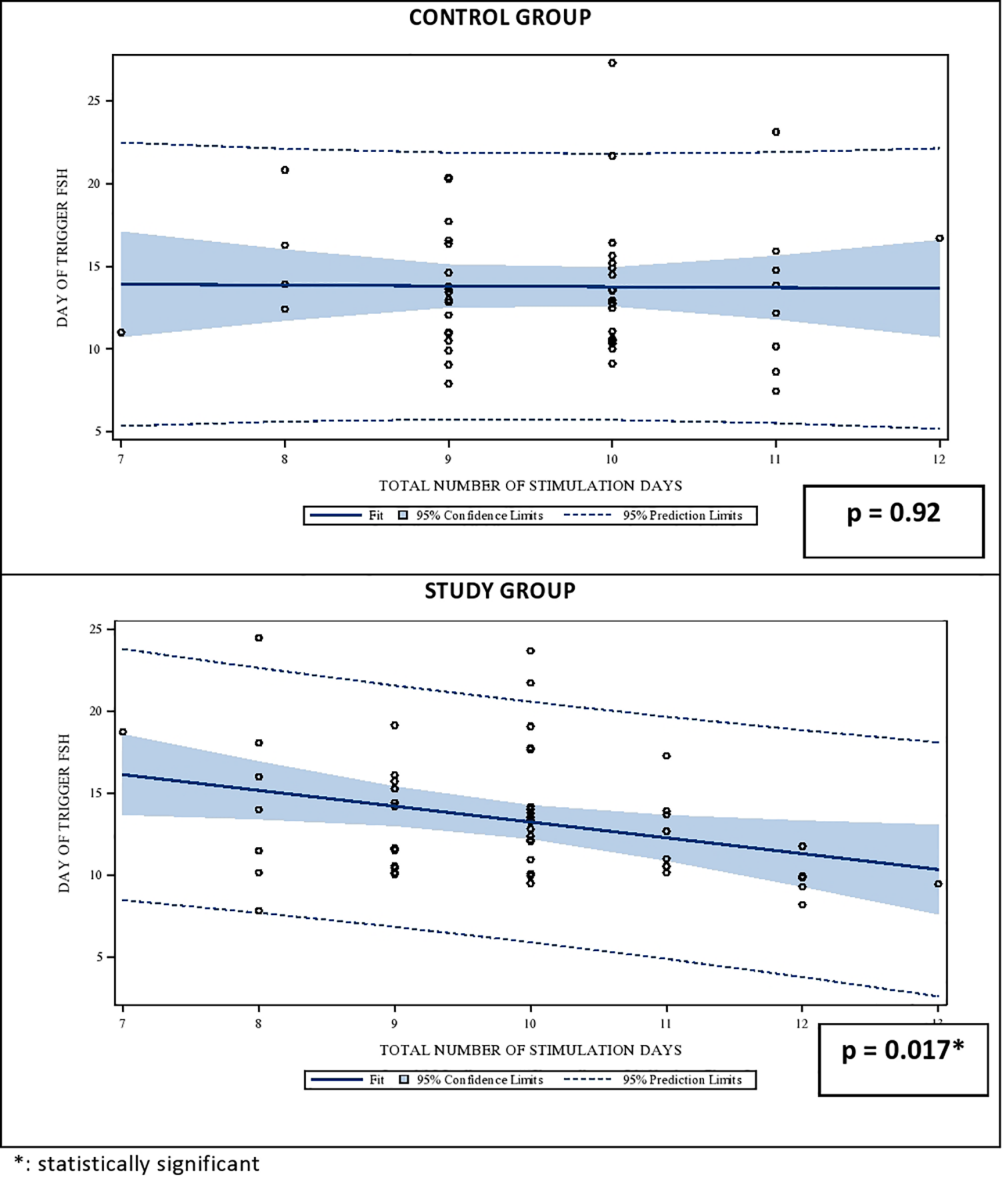
Univariate regression analysis between the number of stimulation days and the median serum FSH level on day of trigger (DoT). FSH, Follicle Stimulating Hormone.

The effect of the gonadotropin dosage reduction on the serum FSH levels on the DoT in the SG becomes obvious in the linear regression analysis between the stimulation duration and the FSH-levels on the DoT. Whereas the FSH-levels remains stable in CG also with a longer stimulation period, the FSH levels in the SG have a statistically significant negative relationship with the number of stimulation days FSH level (p = 0.92 for CG and p = 0.01 for SG, respectively). The correlations are shown in **Figure 1**.

**Figure 2:**
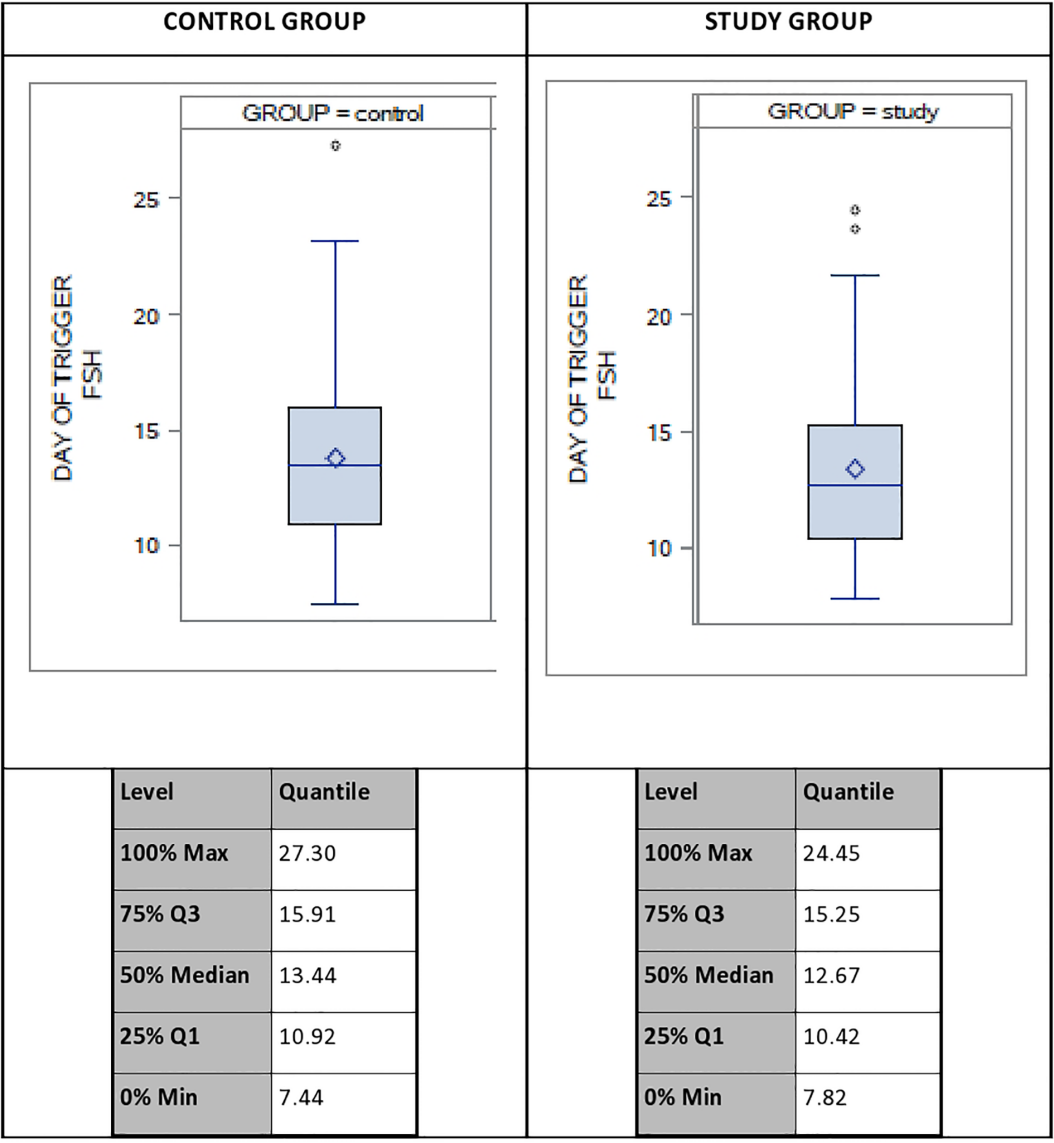
Distribution of systemic FSH-levels on day of trigger (DoT) for control group (CG) and study group (SG). FSH, Follicle Stimulating Hormone.

A descriptive analysis was done to evaluate of the distribution of the systemic FSH levels on DoT in both groups, which revealed a variation of up to 20 IU between the lowest and the highest FSH-levels on the DoT, with a coefficient of variation of 28%. **Figure 2** displays the distribution of the serum FSH levels as well as the FSH levels for the 100%, 75%, 50%, 25% and 0% Percentiles.

To analyze the impact of the patients’ characteristics as well as the different stimulation parameters on the P4- and FSH-level on the DoT, three multivariate analyses were performed due to the fact, that not all parameters could be included into a single multivariate analysis. Analysis 1 included the patient’s characteristics (AMH, AFC, age, BMI, TSH). Analysis 2 included the stimulation parameters total number of follicles on DoT, total Gn-stimulation dosage, total number of stimulation days, DOT-E2- and DOT-FSH-levels and analysis 3 included the number of the follicles of different sizes (< 11 mm, 11 - ≤ 16 mm and ≥ 17 mm) on the DoT (**Tables 4** and **5**).

**Table 4:** Multivariate analysis – correlation of DoT-P4-levels with patient characteristics, stimulation characteristics and follicle size.

Parameter	CG	CG	SG	SG
	p-value	OR-CI 95%	p-value	OR-CI 95%
	**ANALYSIS 1 (PATIENT CHARACTERISTICS)**			
**AMH**	0.42	1.05 (0.94; 1.17)	0.14	1.09 (0.97; 1.23)
**AFC**	0.85	1.00 (0.97; 1.04)	0.88	1.00 (0.97; 1.04)
**Age**	0.14	0.98 (0.95; 1.01)	0.46	1.01 (0.98; 1.04)
**BMI**	0.02*	0.95 (0.92; 0.99)	0.84	1.00 (0.96; 1.05)
**LH Day 2 or 3**	0.91	1.00 (0.96; 1.05)	0.97	1.00 (0.94; 1.06)
	**ANALYSIS 2 (STIMULATION CHARACTERISTICS)**			
**Total Gn-dosage**	0.11	1.00 (1.00; 1.00)	0.22	1.00 (1.00; 1.00)
**Total no of stimulation days**	0.45	1.05 (0.92; 1.20)	0.002*	1.22 (1.08; 1.38)
**DoT-E2-level**	< 0.0001**	1.00 (1.00; 1.00)	0.0001**	1.00 (1.00; 1.00)
**DoT-FSH-level**	0.002*	1.05 (1.02; 1.08)	0.007*	1.05 (1.01; 1.09)
**DoT-LH-level**	0.18	0.93 (0.85; 1.03)	0.54	1.02 (0.95; 1.10)
	**ANALYSIS 3 (FOLLICLE SIZE ON DAY OF TRIGGER)**			
**DoT number of follicles 11-16 mm**	0.19	1.02 (0.99; 1.04)	0.48	1.01 (0.98; 1.04)
**DoT number of follicles ≥ 17 mm**	0.19	1.03 (0.99; 1.07)	0.60	1.01 (0.97; 1.06)

*statistically significant; **highly statistically significant.

CG, Control group; SG, Study group; E2, estradiol; P4, progesterone; FSH, Follicle Stimulating Hormone; LH, Luteinising Hormone; IU, International Units; Gn, Gonadotropin; GnRH, Gonadotropin Releasing Hormone; DoT, day of triggerAMH, Anti-Mullerian-Hormone; BMI, Body Mass Index; DoT, Day of trigger; TSH, Thyroid Stimulating Hormone; Gn, Gonadotropin; E2, estradiol; P4, progesterone; FSH, Follicle Stimulating Hormone.

**Table 5:** Multivariate analysis – correlation of DoT-FSH-levels with patient characteristics, stimulation characteristics and follicle size.

Parameter	CG	CG	SG	SG
	p-value	OR-CI 95%	p-value	OR-CI 95%
	**ANALYSIS 1 (PATIENT CHARACTERISTICS)**			
**AMH**	0.65	0.82 (0.33; 2.01)	0.48	0.72 (0.29; 1.79)
**AFC**	0.02*	0.73 (0.56; 0.96)	0.87	1.02 (0.76; 1.37)
**Age**	0.46	1.08 (0.87; 1.35)	0.61	1.06 (0.85; 1.31)
**BMI**	0.02*	0.69 (0.51; 0.94)	0.60	1.10 (0.85; 1.55)
**LH Day 2 or 3**	0.46	1.15 (0.79; 1.69)	0.24	1.32 (0.83; 2.12)
	**ANALYSIS 2 (STIMULATION CHARACTERISTICS)**			
**Total Gn-dosage**	< 0.0001**	1.01 (1.00; 1.01)	< 0.0001**	1.01 (1.00; 1.01)
**Total no of stimulation days**	0.003*	0.19 (0.07; 0.54)	< 0.0001**	0.11 (0.05; 0.260
**DoT-E2-level**	0.68	1.00 (1.00; 1.00)	0.54	1.00 (1.00; 1.00)
**DoT-P4-level**	0.002*	30.6 (4.05; 40.0)	0.007*	20.01 (2.49; 30.0)
**DoT-LH-level**	0.55	0.77 (0.33; 1.80)	0.43	1.27 (0.27; 2.28)
	**ANALYSIS 3 (FOLLICLE SIZE ON DAY OF TRIGGER)**			
**DoT number of follicles 11-16 mm**	0.09	0.83 (0.67; 1.03)	0.30	0.89 (0.72; 1.11)
**DoT number of follicles ≥ 17 mm**	0.01*	0.65 (0.47; 0.89)	0.06	0.71 (0.50; 1.01)

*statistically significant; **highly statistically significant.

CG, Control group; SG, Study group; E2, estradiol; P4, progesterone; FSH, Follicle Stimulating Hormone; LH, Luteinising Hormone; IU, International Units; Gn, Gonadotropin; GnRH, Gonadotropin Releasing Hormone; DoT, day of trigger; AMH, Anti-Mullerian-Hormone; BMI, Body Mass Index; DoT, Day of trigger; TSH, Thyroid Stimulating Hormone; Gn, Gonadotropin; E2, estradiol; P4, progesterone; FSH, Follicle Stimulating Hormone.

Statistically significant associations for the DoT-P4-level were found for the CG for the BMI (p = 0.01), DoT-E2- and DoT-FSH-level (p = 0.001 each) and for the SG for the total number of stimulation days and the DoT-FSH-level (p = 0.0018; p = 0.0045, respectively) and a highly statistically significant correlation with the DoT-E2-level (p <.0001).

For the DoT-FSH-level, there were statistically significant correlations in the CG between DoT-FSH and AFC (p = 0.02), BMI (p = 0.01), total number of stimulation days (p = 0.0045), DoT-P4-level (p = 0.001) and the DoT number of follicles ≥ 17 mm) and a highly statistically significant correlation with the total Gn-dosage (p <.0001). In the SG, a statistically significant correlation was found with the DoT-P4-level and highly statistically significant correlations with the total Gn-dosage and the total number of stimulation days (both p <.0001).

**Figure 3:**
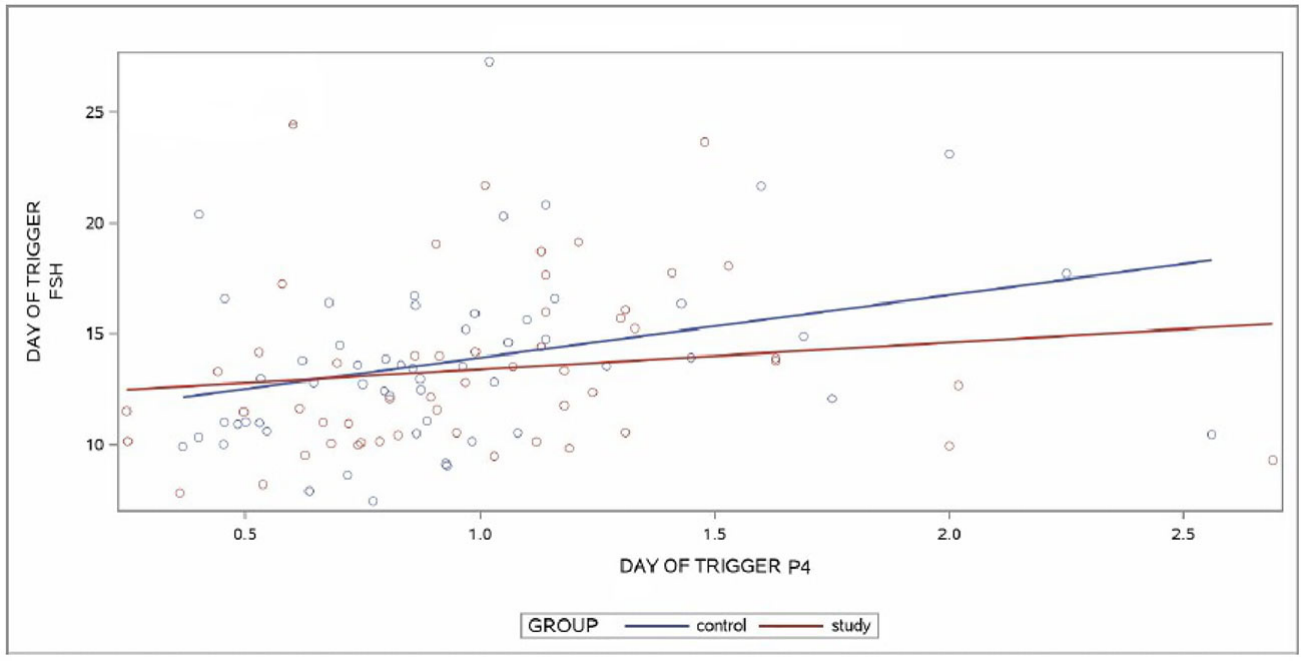
Regression lines of control group (CG) and study group (SG) for the relationship between day of trigger (DoT)-FSH and day of trigger (DoT)-P4 levels. FSH, Follicle Stimulating Hormone; P4, progesterone.

A linear regression model was applied to predict the relationship between DoT-FSH and DoT-P4 levels by group. For the group analysis, a positive linear relationship was found between the two variables in the CG (p = 0.0153) whereas no linear relationship was found between DoT-FSH and DoT-P4 levels in the SG (p = 0.2939). No statistical difference was found when the slopes of the regression lines between CG and SG were compared (p = 0.3806). The slopes are depicted in **Figure 3**.

## Discussion

This study evaluated the impact of a controlled and monitored daily reduction of a small (12.5 IU) rFSH-dosage towards the end of the follicular phase in stimulated IVF/ICSI cycles with the primary aim to show a significant reduction of the P4-level in the study group on the day of final oocyte maturation. Despite the fact that the primary objective of the study was not achieved, the study demonstrated statistically significant correlations between the serum FSH-levels on the day of final oocyte maturation with the progesterone levels (CG: p = 0.001; SG: p = 0.0045) and a positive linear relationship between the serum FSH-DoT- and P4-DoT- levels in the CG (p = 0.0153), whereas no linear relationship was found between these parameters in the SG (p = 0.2939). These findings confirm clearly the influence of enhanced FSH-stimulation on the progesterone levels on the day of final oocyte maturation.

FSH levels above the threshold level during ovarian stimulation are required to initiate and maintain multifollicular growth (20) and due to the half lifetime of exogenously administered gonadotropins (21, 22), rec-FSH injections have to be administered on a daily basis and usually the gonadotropin dosage remains unchanged until the day of final oocyte maturation. It is well described on a molecular level (7), that FSH stimulates the expression of 3β-hydroxysteroid dehydrogenase (3β-HSD) and progesterone biosynthesis in human granulosa cells in addition to its stimulatory effect on the expression of other steroidogenic enzymes, required for estrogen synthesis. Due to the direct stimulatory effect of FSH on the enzymatic activity of 3β-HSD, the conversion of pregnenolone to progesterone is increased, leading to an enhanced progesterone and estradiol output, in a dose-dependent fashion. Following this physiological mechanism, a step-down protocol, using a higher reduction dosage, might have the potential to avert premature progesterone rise and therefore gain importance in the context of prevention of this event. So far, step-down protocols are described in anovulatory patients (WHO group II anovulation) for ovulation induction (23) and in ovarian stimulation for IVF/ICSI they are mainly used in high-responder patients to prevent ovarian hyperstimulation syndrome (OHSS), in patients with polycystic ovarian syndrome (PCOS) or to mimic the physiologic FSH course of a natural cycle in an ovarian stimulation cycle (24, 25).

Studies evaluating the endocrine profile during ovarian stimulation have shown a rise in serum FSH levels until day 6 of stimulation, thereafter they remain constant, when the exogenous rec-FSH dosage is not altered (26, 27). As expected, the current data revealed a rise in serum FSH-levels after stimulation initiation. In the further stimulation course of the SG, serum FSH levels remained constant from the start of the Gn-dosage reduction until the day of trigger, despite a stepwise reduction of daily 12.5 IU over a median time period of 3 days. Gonal f^®^, which was used in the herein presented study as gonadotropin for stimulation, has a half lifetime of about 42.58 h after a single injection of 225 IU (21). Daily injections lead to an accumulation of exogenous gonadotropins and therefore explain not only the lack of a decrease in FSH serum levels within the three-day period of Gn-reduction in the SG, but also that no difference was seen in the FSH serum levels on DoT between the SG and the CG. The fact, that there was no difference in the stimulation outcome regarding the number of retrieved and mature oocytes between the patients with and without gonadotropin dosage reduction demonstrates that intense exogenous FSH stimulation might not be required once a follicle size of 14 mm and beyond has been achieved.

For follicle growth, steroid hormone production and oocyte maturation, FSH as well as LH are crucial, however, with a different impact throughout the menstrual cycle. At the early antral follicle stage, granulosa cells (GC) are sensitive to FSH stimulation. Besides FSHR (FSH receptor), also the LH receptor (LHR) becomes expressed on GC and contributes to the production of progesterone and estradiol by the follicle (28). Induction of LHR expression on GC is mainly regulated by FSH, and as the follicle matures into the preovulatory stage, LHR induction occurs more effectively (29). The number of FSHR and LHR changes with the follicle growth with a decline of the FSHR and a parallel increase of the LHR. This shift in the receptor numbers occurs from a follicle size of approximately 15 mm and indicates that follicles lose responsiveness to FSH and acquire responsiveness to LH as they progress through the follicular phase (30). In a natural cycle, this shift from FSHR to LHR is the basis of the continuous growth of the selected, dominant follicle despite the progressive fall of the FSH serum concentration in the late follicular phase. These changes in the receptor predominance represent the rationale of our findings, that a small reduction of the FSH stimulation dosage from a follicle size of 14 mm onwards does not adversely impact the further follicle development and the oocyte yield.

Additionally, this study revealed that patients metabolize exogenous FSH administration differently as our analysis of serum FSH levels on the day of final oocyte maturation clearly demonstrated. A clinically considerable discrepancy between the lowest and the highest serum FSH levels was identified in both groups with a difference of up to 20 IU. Despite the fact, that the multivariate analysis did not reveal a difference when the stimulation parameters were correlated with the serum FSH levels between the groups, these results raise the question of the “adequate” serum FSH level for ovarian stimulation, especially with the knowledge of a reduced responsiveness of the granulosa cells towards exogenous FSH following a decrease of FSH receptors.

In conclusion, this study revealed basic insights into the physiology of ovarian stimulation. The fact, that the primary aim of a progesterone reduction by a step-down of 12.5 IU recFSH could not be achieved can be seen as a limitation. However, the data confirmed that enhanced FSH stimulation is a source of progesterone on the day of final oocyte maturation and following the pathophysiology, the use of an increased step-down dosage should accomplish this goal. The strength of our study lies in the finding that Gn-reduction in small steps from a follicle size of 14 mm onwards does not lead to a decrease in the systemic FSH serum levels and does not have an impact on the number of retrieved and mature oocytes.

Future studies should investigate the minimal serum FSH blood levels required in the late follicular phase to support further follicle growth and, at the same time, prevent premature progesterone elevation, but also should evaluate to possibility to perform “FSH-level-based” individualized ovarian stimulation according to the patients’ specific needs.

## Data Availability Statement

The original contributions presented in the study are included in the article/supplementary material. Further inquiries can be directed to the corresponding author.

## Ethics Statement

The studies involving human participants were reviewed and approved by the ethics committee of the IVIRMA Abu Dhabi Fertility Clinic, Abu Dhabi, UAE (approval number: REFA012) (now ART Fertility Clinic Abu Dhabi, UAE) and was registered with clinicaltrials.gov. under the number NCT03356964. The patients/participants provided their written informed consent to participate in this study.

## Author Contributions

BL: Conceptualization of study, patient recruitment, data analysis, drafting of paper. CC: recruitment of patients, linguistic review of paper. LM: recruitment of patients, review of paper. SD: data recording, data management. JS: hormonal analysis of blood samples. AJ: hormonal analysis of blood samples. HF: Conceptualization of study, patient recruitment, review of paper. All authors contributed to the article and approved the submitted version.

## Funding

This research was financially supported by Merck Serono Middle East FZ-LTD, an affiliate of Merck KGaA, Darmstadt, Germany. Merck KGaA, Darmstadt, Germany, reviewed the manuscript for medical accuracy only before journal submission. The authors are fully responsible for the content of this manuscript, and the views and opinions described in the publication reflect solely those of the authors.

## Conflict of Interest

The authors declare that the study “Impact on reduction of step-down- approach during late follicular phase in recombinant FSH-stimulation dosage for IVF on Progesterone level” received funding from Merck Serono Middle East FZ-LTD, an affiliate of Merck KGaA, Darmstadt, Germany. The funder, Merck KGaA, Darmstadt, Germany, had no role in study design, data collection and analysis, decision to publish, or preparation of the manuscript and reviewed the manuscript for medical accuracy only before journal submission with the study.
